# Bis[*trans*-dichloridobis(propane-1,3-diamine-κ^2^ *N*,*N*′)chromium(III)] tetra­chloridozincate determined using synchrotron radiation

**DOI:** 10.1107/S1600536812023355

**Published:** 2012-05-26

**Authors:** Dohyun Moon, Md Abdus Subhan, Jong-Ha Choi

**Affiliations:** a Pohang Accelerator Laboratory, POSTECH, Pohang 790-784, Republic of Korea; b Department of Chemistry, Shah Jalal University of Science and Technology, Sylhet, Bangladesh; c Department of Chemistry, Andong National University, Andong 760-749, Republic of Korea

## Abstract

In the title compound, [CrCl_2_(C_3_H_10_N_2_)_2_]_2_[ZnCl_4_], the Cr^III^ atom is coordinated by four N atoms of propane-1,3-diamine (tn) and two Cl atoms in a *trans* arrangement, displaying a distorted octa­hedral geometry with crystallographic inversion symmetry; the Zn atom in the [ZnCl_4_]^2−^ anion lies on a -4 axis. The orientations of the two six-membered chelate rings in the complex cation are in an *anti* chair–chair conformation with respect to each other. The Cr—N bond lengths are 2.087 (6) and 2.097 (6) Å. The Cr—Cl and Zn—Cl bond lengths are 2.3151 (16) and 2.3255 (13) Å, respectively. Weak inter­molecular hydrogen bonds involving the tn NH_2_ groups as donors and chloride ligands of the anion and cation as acceptors are observed.

## Related literature

For the synthesis, see: House (1970 ▶). For the structures of *trans*-[Cr(tn)_2_ *L*_2_]ClO_4_ (*L* = F, Cl, Br), see: Vaughn & Rogers (1985 ▶); Choi & Clegg (2011 ▶); Choi *et al.* (2012 ▶). For the structures of *trans*-[Cr(Me_2_tn)_2_Cl_2_]ClO_4_ and *trans*-[Cr(Me_2_tn)_2_Cl_2_]_2_ZnCl_4_, see: Choi *et al.* (2008 ▶, 2011 ▶).
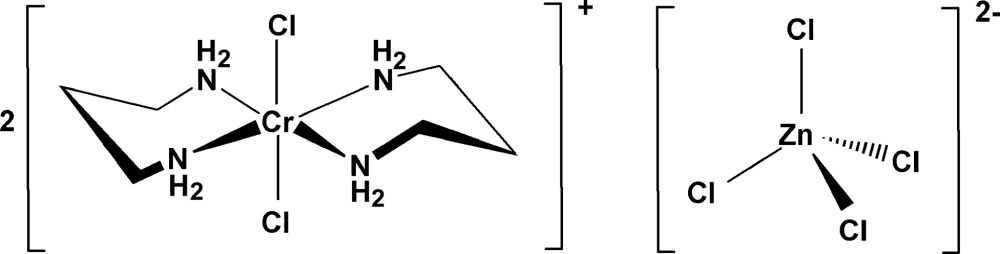


## Experimental

### 

#### Crystal data

[CrCl_2_(C_3_H_10_N_2_)_2_]_2_[ZnCl_4_]
*M* *_r_* = 749.49
Tetragonal, 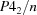

*a* = 15.141 (2) Å
*c* = 6.4220 (13) Å
*V* = 1472.2 (4) Å^3^
*Z* = 2
Synchrotron radiation, λ = 0.90000 Å
μ = 4.26 mm^−1^
*T* = 95 K
0.16 × 0.02 × 0.02 mm

#### Data collection

ADSC Q210 CCD area-detector diffractometer
Absorption correction: multi-scan (*HKL-3000* *SCALEPACK*; Otwinowski & Minor, 1997 ▶) *T*_min_ = 0.549, *T*_max_ = 0.920
7735 measured reflections
1225 independent reflections
1157 reflections with *I* > 2σ(*I*)
*R*_int_ = 0.030

#### Refinement

*R*[*F* ^2^ > 2σ(*F* ^2^)] = 0.066
*wR*(*F* ^2^) = 0.198
*S* = 1.11
1225 reflections
73 parameters
H-atom parameters constrained
Δρ_max_ = 2.55 e Å^−3^
Δρ_min_ = −1.30 e Å^−3^

### 

Data collection: *PAL ADSC Quantum-210 ADX Program* (Arvai & Nielsen, 1983 ▶); cell refinement: *HKL-3000* (Otwinowski & Minor, 1997 ▶); data reduction: *HKL-3000*; program(s) used to solve structure: *SHELXTL* (Sheldrick, 2008 ▶); program(s) used to refine structure: *SHELXTL*; molecular graphics: *DIAMOND* (Brandenburg, 2012 ▶); software used to prepare material for publication: *WinGX* (Farrugia, 1999 ▶).

## Supplementary Material

Crystal structure: contains datablock(s) I, global. DOI: 10.1107/S1600536812023355/nk2158sup1.cif

Structure factors: contains datablock(s) I. DOI: 10.1107/S1600536812023355/nk2158Isup2.hkl

Additional supplementary materials:  crystallographic information; 3D view; checkCIF report

## Figures and Tables

**Table 1:** Hydrogen-bond geometry (Å, °)

*D*—H⋯*A*	*D*—H	H⋯*A*	*D*⋯*A*	*D*—H⋯*A*
N1—H1*B*⋯Cl2	0.92	2.90	3.678 (6)	144
N1—H1*A*⋯Cl2^i^	0.92	2.87	3.636 (6)	142
N1—H1*B*⋯Cl2^ii^	0.92	2.82	3.529 (6)	134
N2—H2*A*⋯Cl2^iii^	0.92	2.72	3.641 (6)	179
N2—H2*B*⋯Cl1^iv^	0.92	2.56	3.404 (6)	154

Symmetry codes: (i) 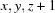; (ii) 
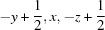
; (iii) 
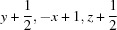
; (iv) 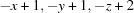.

**Fig. 1.**
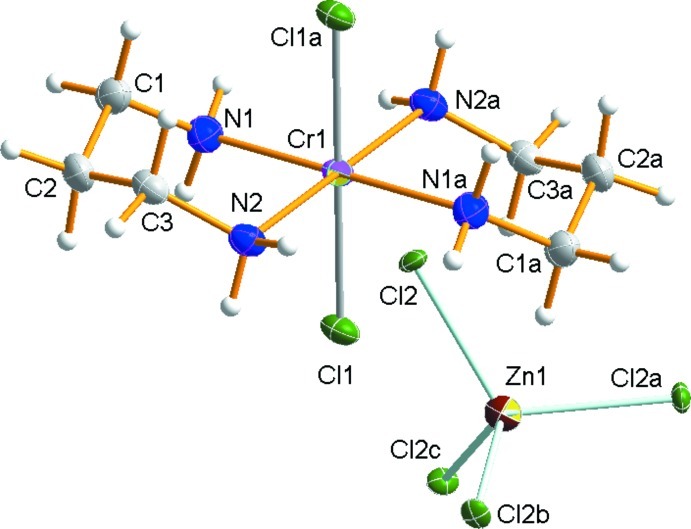
A perspective drawing (30% probability level) of the one complex cation and anion

## supplementary crystallographic information

### Comment

There are two possible conformations with respect to the six-membered rings in the *trans*-[Cr(tn)_2_*L*_2_]^+^(tn = propane-1,3-diamine, *L* = monodentate). The carbon atoms of the two chelate rings of the tn ligands can be located on the same side (*syn* conformer) or on opposite side (*anti* conformer) of the equatorial plane. The preference for *syn*- or *anti*-conformation in the complex cation is still of interest. The infrared and electronic absorption spectroscopic methods are not useful in distinguishing the *syn* or *anti* conformaions of the six-membered chelate rings in the typed transition metal complexes. The different arrangements of the two six-membered chelate rings of tn ligands may be dependent on the packing forces and counter anions in the crystal structure.

In this communication, we report the structure of *trans*-[Cr(tn)_2_Cl_2_]_2_ZnCl_4_ in order to obtain more information on the conformation of the two six-membered chelate rings.

Counter anionic species play a very important role in coordination chemistry. This is another example of a *trans*-[Cr(tn)_2_Cl_2_]^+^ but with different counter anion (Choi *et al.*, 2008). The structural analysis shows that there is only one crystallographically independent Cr^III^ complex cation where the four nitrogen atoms of two tn ligands occupy the equatorial sites and the two chlorine atoms coordinate to the Cr metal centre in *trans* configuration. The Cr1 moiety of complex cation is half occupancy in the asymmetric unit. An ellipsoid plot (30% probability level) of the one Cr^III^ complex cation and anion in the title compound, together with the atomic labelling, is depicted in Fig. 1.

The two six-membered rings have stable chair conformations. Atom Cr1 is located at a crystallographic centre of symmetry, so the Cr complex cation has molecular *C_i_* symmetry. The two chelate rings in the Cr complex cation adopt same *anti* chair-chair conformation with respect to each other. The Cr—N(tn) distances of 2.087 (76) and 2.097 (6) Å are good agreement with average Cr—N distance of 2.0884 (19) Å found in *trans*-[Cr(tn)_2_Cl_2_]ClO_4_ (Choi & Clegg, 2011), and the range of 2.0741 (19) to 2.0981 (18) Å found in *trans*-[Cr(Me_2_tn)_2_Cl_2_]_2_ZnCl_4_ (Me_2_tn = 2,2-dimethylpropane-1,3-diamine) (Choi, Joshi & Spiccia, 2011). The Cr–Cl distance of 2.315 (2) Å is longer than the 2.085 (4) Å of Cr—F found in *trans*-[Cr(tn)_2_F_2_]ClO_4_ (Vaughn & Rogers, 1985), but slightly shorter than 2.4681 (4) Å of Cr—Br *trans*-[Cr(tn)_2_Br_2_]ClO_4_ (Choi *et al.*, 2012). The other N—C and C—C bond distances and Cr—N—C, N—C—C and C—C—C angles are also of usual values for tn ligands in chair conformations. The crystals are held together by weak hydrogen bonds (Table 1) between the NH groups of the tn, Cl ligand and Cl atom of tetrachlorozincate anion. The uncoordinated ZnCl_4_^2-^ anion from chromium(III) ion remains outside the coordination sphere. As expected, the Zn atom in the ZnCl_4_^2-^ has a tetrahedral coordination surrounded by four Cl atoms. The Zn—Cl bond distance of 2.3255 (13) and the Cl—Zn—Cl angles of 106.28 (4)–116.05 (9)° are observed. The coordinate Cl1 atom in Cr1 complex cation also forms one hydrogen bond with one NH group in the neighbouring complex cation. The differences found in the conformations of the two six-membered chelate rings may be attributed to the differences in the hydrogen bonding networks and crystal packing forces between the chromium(III) complex cation and counter anion.

### Experimental

The propane-1,3-diamine was obtained from Aldrich Chemical Co. and used as supplied. All chemicals were reagent grade materials and used without further purification. As starting materials, *trans*-[Cr(tn)_2_Cl_2_]ClO_4_ was prepared as described in the literature (House, 1970; Choi & Clegg, 2011). The crude chloride salt (0.75 g) was dissolved in 20 ml of 0.1*M* HCl at 40°C and added 10 ml of 6*M* HCl containing 1.2 g of solid ZnCl_2_. The resulting solution was filtered and allowed to stand at room temperature for several days to give green crystals of the tetrachlorozincate(II) salt suitable for X-ray structural analysis.

### Refinement

Non-hydrogen atoms were refined anisotropically; hydrogen atoms were first located in a difference map; N–H hydrogen atoms were freely refined and C–H hydrogen atoms were constrained to ride on the parent carbon atom, with C–H = 0.98 Å and C–H = 0.99 Å and *U*_iso_(H) = 1.2*U*_eq_(C) for methylene groups.

### Crystal data

**Table:**

[CrCl_2_(C_3_H_10_N_2_)_2_]_2_[ZnCl_4_]	*D*_x_ = 1.691 Mg m^−^^3^
*M**_r_* = 749.49	Synchrotron radiation, λ = 0.90000 Å
Tetragonal, *P*4_2_/*n*	Cell parameters from 16475 reflections
*a* = 15.141 (2) Å	θ = 1.0–33.7°
*c* = 6.4220 (13) Å	µ = 4.26 mm^−^^1^
*V* = 1472.2 (4) Å^3^	*T* = 95 K
*Z* = 2	Needle, pale blue
*F*(000) = 764	0.16 × 0.02 × 0.02 mm

### Data collection

**Table:**

ADSC Q210 CCD area-detector diffractometer	1225 independent reflections
Radiation source: PLSII 2D bending magnet	1157 reflections with *I* > 2σ(*I*)
Si(111) double crystal monochromator	*R*_int_ = 0.030
ω and kappa scan	θ_max_ = 32.0°, θ_min_ = 2.4°
Absorption correction: multi-scan (*HKL-3000**SCALEPACK*; Otwinowski & Minor, 1997)	*h* = −17→17
*T*_min_ = 0.549, *T*_max_ = 0.920	*k* = −17→17
7735 measured reflections	*l* = −7→7

### Refinement

**Table:**

Refinement on *F*^2^	Secondary atom site location: difference Fourier map
Least-squares matrix: full	Hydrogen site location: inferred from neighbouring sites
*R*[*F*^2^ > 2σ(*F*^2^)] = 0.066	H-atom parameters constrained
*wR*(*F*^2^) = 0.198	*w* = 1/[σ^2^(*F*_o_^2^) + (0.1185*P*)^2^ + 13.7893*P*] where *P* = (*F*_o_^2^ + 2*F*_c_^2^)/3
*S* = 1.11	(Δ/σ)_max_ < 0.001
1225 reflections	Δρ_max_ = 2.55 e Å^−^^3^
73 parameters	Δρ_min_ = −1.30 e Å^−^^3^
0 restraints	Extinction correction: *SHELXTL* (Sheldrick, 2008), Fc^*^=kFc[1+0.001xFc^2^λ^3^/sin(2θ)]^-1/4^
Primary atom site location: structure-invariant direct methods	Extinction coefficient: 0.010 (2)

### Special details

**Table:**

Geometry. All e.s.d.'s (except the e.s.d. in the dihedral angle between two l.s. planes) are estimated using the full covariance matrix. The cell e.s.d.'s are taken into account individually in the estimation of e.s.d.'s in distances, angles and torsion angles; correlations between e.s.d.'s in cell parameters are only used when they are defined by crystal symmetry. An approximate (isotropic) treatment of cell e.s.d.'s is used for estimating e.s.d.'s involving l.s. planes.
Refinement. Refinement of *F*^2^ against ALL reflections. The weighted *R*-factor *wR* and goodness of fit *S* are based on *F*^2^, conventional *R*-factors *R* are based on *F*, with *F* set to zero for negative *F*^2^. The threshold expression of *F*^2^ > σ(*F*^2^) is used only for calculating *R*-factors(gt) *etc*. and is not relevant to the choice of reflections for refinement. *R*-factors based on *F*^2^ are statistically about twice as large as those based on *F*, and *R*- factors based on ALL data will be even larger.

### Fractional atomic coordinates and isotropic or equivalent isotropic displacement parameters (Å^2^)

**Table:**

	*x*	*y*	*z*	*U*_iso_*/*U*_eq_	
Cr1	0.5000	0.5000	0.5000	0.0176 (6)	
Cl1	0.42602 (11)	0.55586 (11)	0.7867 (2)	0.0219 (6)	
N1	0.4440 (4)	0.3768 (4)	0.5622 (10)	0.0250 (14)	
H1A	0.4141	0.3811	0.6865	0.030*	
H1B	0.4027	0.3657	0.4605	0.030*	
N2	0.6077 (4)	0.4700 (4)	0.6928 (9)	0.0214 (13)	
H2A	0.6528	0.5081	0.6592	0.026*	
H2B	0.5914	0.4822	0.8277	0.026*	
C1	0.5033 (5)	0.2982 (5)	0.5752 (11)	0.0249 (16)	
H1C	0.5309	0.2880	0.4375	0.030*	
H1D	0.4676	0.2454	0.6100	0.030*	
C2	0.5748 (5)	0.3093 (5)	0.7361 (11)	0.0244 (16)	
H2C	0.6049	0.2518	0.7544	0.029*	
H2D	0.5465	0.3245	0.8705	0.029*	
C3	0.6441 (5)	0.3786 (5)	0.6887 (11)	0.0240 (15)	
H3A	0.6923	0.3740	0.7922	0.029*	
H3B	0.6696	0.3669	0.5494	0.029*	
Zn1	0.2500	0.2500	0.2500	0.0223 (6)	
Cl2	0.37601 (8)	0.28336 (9)	0.0585 (2)	0.0115 (5)	

### Atomic displacement parameters (Å^2^)

**Table:**

	*U*^11^	*U*^22^	*U*^33^	*U*^12^	*U*^13^	*U*^23^
Cr1	0.0199 (9)	0.0192 (9)	0.0138 (9)	−0.0007 (5)	−0.0008 (6)	−0.0002 (6)
Cl1	0.0230 (9)	0.0287 (10)	0.0141 (9)	0.0025 (6)	0.0014 (6)	−0.0007 (6)
N1	0.026 (3)	0.024 (3)	0.025 (3)	0.000 (2)	−0.001 (3)	0.002 (3)
N2	0.024 (3)	0.024 (3)	0.016 (3)	−0.002 (2)	0.000 (2)	−0.001 (2)
C1	0.031 (4)	0.020 (3)	0.023 (4)	0.001 (3)	−0.002 (3)	−0.002 (3)
C2	0.027 (4)	0.021 (3)	0.025 (4)	−0.001 (3)	−0.002 (3)	0.004 (3)
C3	0.021 (3)	0.029 (4)	0.022 (3)	0.002 (3)	−0.002 (3)	0.003 (3)
Zn1	0.0223 (7)	0.0223 (7)	0.0222 (9)	0.000	0.000	0.000
Cl2	0.0087 (8)	0.0138 (8)	0.0119 (8)	−0.0024 (5)	0.0055 (5)	−0.0009 (5)

### Geometric parameters (Å, º)

**Table:**

Cr1—N1	2.087 (6)	C1—C2	1.505 (10)
Cr1—N1^i^	2.087 (6)	C1—H1C	0.9900
Cr1—N2	2.097 (6)	C1—H1D	0.9900
Cr1—N2^i^	2.097 (6)	C2—C3	1.515 (10)
Cr1—Cl1^i^	2.3151 (16)	C2—H2C	0.9900
Cr1—Cl1	2.3151 (16)	C2—H2D	0.9900
N1—C1	1.494 (9)	C3—H3A	0.9900
N1—H1A	0.9200	C3—H3B	0.9900
N1—H1B	0.9200	Zn1—Cl2^ii^	2.3255 (13)
N2—C3	1.489 (9)	Zn1—Cl2^iii^	2.3255 (13)
N2—H2A	0.9200	Zn1—Cl2^iv^	2.3255 (13)
N2—H2B	0.9200	Zn1—Cl2	2.3255 (13)
			
N1—Cr1—N1^i^	179.999 (1)	H2A—N2—H2B	107.1
N1—Cr1—N2	90.5 (2)	N1—C1—C2	112.4 (6)
N1^i^—Cr1—N2	89.5 (2)	N1—C1—H1C	109.1
N1—Cr1—N2^i^	89.5 (2)	C2—C1—H1C	109.1
N1^i^—Cr1—N2^i^	90.5 (2)	N1—C1—H1D	109.1
N2—Cr1—N2^i^	179.999 (1)	C2—C1—H1D	109.1
N1—Cr1—Cl1^i^	91.27 (18)	H1C—C1—H1D	107.9
N1^i^—Cr1—Cl1^i^	88.73 (18)	C1—C2—C3	115.9 (6)
N2—Cr1—Cl1^i^	90.80 (16)	C1—C2—H2C	108.3
N2^i^—Cr1—Cl1^i^	89.20 (16)	C3—C2—H2C	108.3
N1—Cr1—Cl1	88.73 (18)	C1—C2—H2D	108.3
N1^i^—Cr1—Cl1	91.27 (18)	C3—C2—H2D	108.3
N2—Cr1—Cl1	89.20 (16)	H2C—C2—H2D	107.4
N2^i^—Cr1—Cl1	90.80 (16)	N2—C3—C2	112.5 (6)
Cl1^i^—Cr1—Cl1	180.0	N2—C3—H3A	109.1
C1—N1—Cr1	118.6 (4)	C2—C3—H3A	109.1
C1—N1—H1A	107.7	N2—C3—H3B	109.1
Cr1—N1—H1A	107.7	C2—C3—H3B	109.1
C1—N1—H1B	107.7	H3A—C3—H3B	107.8
Cr1—N1—H1B	107.7	Cl2^ii^—Zn1—Cl2^iii^	106.24 (3)
H1A—N1—H1B	107.1	Cl2^ii^—Zn1—Cl2^iv^	106.25 (3)
C3—N2—Cr1	118.6 (4)	Cl2^iii^—Zn1—Cl2^iv^	116.14 (7)
C3—N2—H2A	107.7	Cl2^ii^—Zn1—Cl2	116.14 (7)
Cr1—N2—H2A	107.7	Cl2^iii^—Zn1—Cl2	106.24 (3)
C3—N2—H2B	107.7	Cl2^iv^—Zn1—Cl2	106.25 (3)
Cr1—N2—H2B	107.7		

Symmetry codes: (i) −*x*+1, −*y*+1, −*z*+1; (ii) −*x*+1/2, −*y*+1/2, *z*; (iii) *y*, −*x*+1/2, −*z*+1/2; (iv) −*y*+1/2, *x*, −*z*+1/2.

### Hydrogen-bond geometry (Å, º)

**Table:**

*D*—H···*A*	*D*—H	H···*A*	*D*···*A*	*D*—H···*A*
N1—H1*B*···Cl2	0.92	2.90	3.678 (6)	144
N1—H1*A*···Cl2^v^	0.92	2.87	3.636 (6)	142
N1—H1*B*···Cl2^iv^	0.92	2.82	3.529 (6)	134
N2—H2*A*···Cl2^vi^	0.92	2.72	3.641 (6)	179
N2—H2*B*···Cl1^vii^	0.92	2.56	3.404 (6)	154

Symmetry codes: (iv) −*y*+1/2, *x*, −*z*+1/2; (v) *x*, *y*, *z*+1; (vi) *y*+1/2, −*x*+1, *z*+1/2; (vii) −*x*+1, −*y*+1, −*z*+2.
